# Strengthening the science of addressing antimicrobial resistance: a framework for planning, conducting and disseminating antimicrobial resistance intervention research

**DOI:** 10.1186/s12961-020-00549-1

**Published:** 2020-06-08

**Authors:** S. Rogers Van Katwyk, S. J. Hoffman, M. Mendelson, M. Taljaard, J. M. Grimshaw

**Affiliations:** 1 School of Epidemiology and Public Health, University of Ottawa, Ottawa, ON Canada; 2 Global Strategy Lab, Dahdaleh Institute for Global Health Research, Faculty of Health and Osgoode Hall Law School, York University, Toronto, ON Canada; 3 Department of Health Research Methods, Evidence, and Impact and McMaster Health Forum, McMaster University, Hamilton, ON Canada; 4 Department of Global Health & Population, Harvard T.H. Chan School of Public Health, Harvard University, Boston, MA United States of America; 5 Division of Infectious Diseases and HIV Medicine, Groote Schuur Hospital, University of Cape Town, Cape Town, South Africa; 6 Clinical Epidemiology Program, Ottawa Hospital Research Institute, Ottawa, ON Canada; 7 Department of Medicine, University of Ottawa, Ottawa, ON Canada

**Keywords:** Antimicrobial resistance, evidence-informed policy, health policy, evaluation

## Abstract

Antimicrobial resistance (AMR) has the potential to threaten tens of millions of lives and poses major global economic and development challenges. As the AMR threat grows, it is increasingly important to strengthen the scientific evidence base on AMR policy interventions, to learn from existing policies and programmes, and to integrate scientific evidence into the global AMR response.

While rigorous evaluations of AMR policy interventions are the ideal, they are far from the current reality. To strengthen this evidence base, we describe a framework for planning, conducting and disseminating research on AMR policy interventions. The framework identifies challenges in AMR research, areas for enhanced coordination and cooperation with decision-makers, and best practices in the design of impact evaluations for AMR policies.

This framework offers a path forward, enabling increased local and global cooperation, and overcoming common limitations in existing research on AMR policy interventions.

## Introduction

Antimicrobial resistance (AMR) — the process by which microbes acquire resistance to antimicrobial medicines — is widely recognised as a serious threat to global public health. The likelihood of drug resistance increases when microbes are exposed to antimicrobials and, unlike previous generations, we can no longer count on the development of new drugs to overcome this threat [1–3]. The development of resistance has been accelerated by overuse of antimicrobials for medical and agricultural purposes. AMR now threatens tens of millions of lives [4], in addition to posing major global economic and development challenges [5].

AMR is politically, economically and microbially difficult to tackle from a policy perspective. Efforts to evaluate AMR interventions would be significantly improved by increasing investments in monitoring and surveillance for antimicrobial resistance. Controlling AMR will require a suite of effective antimicrobial stewardship and conservation strategies to ensure the appropriate use of antimicrobials [6], in addition to substantial international cooperation on the regulation and surveillance of antimicrobials and their use [7–11]. Substantial research is needed to generate evidence on the effects and effectiveness of various possible AMR policies and to ensure that health system investments in AMR are evidence informed. Existing research has created little clarity about what interventions are best suited to achieve AMR goals across contexts, cultures and health systems. Many efforts to reduce AMR are designed as policies to reduce the use of antimicrobials; in this manuscript, when we refer to ‘AMR policy’, we refer also to these antimicrobial use policies. Policy recommendations for AMR have changed little since the late 1990s [4, 12–16]. Worldwide, millions of dollars are invested annually in public programmes to raise awareness about AMR, educate health professionals on appropriate prescribing, and decrease antimicrobial consumption in the health and agricultural sectors. Despite major financial and political investments, it has been difficult to link these programmes to concrete improvements in antimicrobial use, resistance or health outcomes more generally [6, 17], particularly as major surveillance and information gaps impede the global response to AMR [18] (Box 1).

**Fig. 1:**
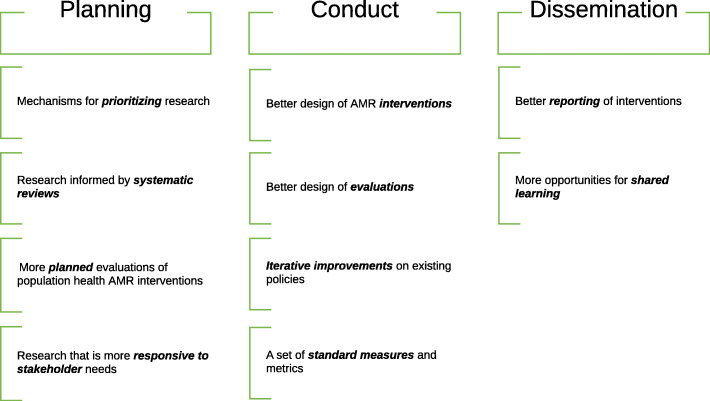
Framework for prioritising, conducting and disseminating AMR policy interventions

As the threat posed by AMR grows, it is increasingly important to strengthen the scientific evidence base on AMR policy interventions, to learn from existing policies and programmes, and integrate scientific evidence into the global AMR response [6]. The goal of this paper is to develop a framework that facilitates the strengthening of this evidence base. This paper is not intended as a formal research prioritisation process but, rather, builds upon the findings from recent systematic reviews of interventions to reduce antimicrobial consumption [6, 17, 21, 25] and efforts by others to strengthen research on AMR and public health [17, 23, 26–30], and aims to draw insights for improving the planning, conduct and dissemination of research to evaluate AMR policy interventions (Fig. 1). The framework identifies challenges in AMR evaluation research, areas for enhanced coordination and cooperation with policy-makers, and best-practices for overcoming common limitations in evaluating AMR policies. Some of these challenges are specific to AMR, while others are shared with other areas of health research.

### Research planning

#### Prioritising research

AMR researchers need to prioritise the study of ‘what’ works, ‘when’ it works, ‘why’ it works, and ‘what’ elements are necessary for its success.

AMR needs better mechanisms for prioritising important research questions that can shape effective action. There has already been substantial research on the root social and microbial causes of AMR [31–33] and we argue that attention now needs to be focused on determining which interventions are effective at addressing the underlying root causes of AMR, why these interventions work, what elements are necessary to their success, and in what contexts and circumstances these interventions work. More evidence on all four questions would be invaluable for policy planning [6, 17]. As the majority of existing research evidence focuses on interventions in high-income settings [6, 17], additional research on these questions would particularly benefit low- and middle-income countries (LMICs) and other resource limited settings to identify policy interventions that can be adapted to meet local needs and priorities.

More formally, research prioritisation can be improved by undertaking structured prioritisation and consensus processes in collaboration with stakeholder groups, including policy-makers at different levels of government, civil society, health professionals and research funders. Research funders can support this work through opportunities for strategically funded research to address AMR rather than relying on researcher-led operating grants. For example, the Joint Programming Initiative on Antimicrobial Resistance (JPIAMR) recently funded an international workshop and formal consensus process to develop 10 research priorities for behavioural approaches to develop more impactful hospital antimicrobial stewardship programmes [29]. The James Lind Alliance in the United Kingdom, which provides a platform for priority-setting partnerships between clinicians, patients and carers, has produced a guidebook that outlines their method for identifying research uncertainties and producing an agreed list of research priorities [34]. Research prioritisation processes can also highlight the differences between research priorities in different contexts. Economic, political and cultural differences between countries and regions are likely to introduce new priorities. A recent prioritisation exercise looking at global health trial methodology found different research priorities in LMICs compared with the United Kingdom [35]. The Cochrane Collaboration has previously published a special series on priority-setting that offers guidance on topics such as applying an equity lens to priority-setting [36] and effective stakeholder participation in priority-setting [37]. Formal prioritisation processes would help drive research agendas at the international level, such as those of JPIAMR, WHO, the Food and Agriculture Organization of the United Nations, and the World Organisation for Animal Health. Recently, the WHO has engaged in formal priority-setting exercises for AMR research and development, first through a multi-criteria decision analysis exercise to develop its list of priority pathogens for research and development of new antimicrobials [38], and more recently through the Global Antibiotic Research & Development Partnership (GARDP). At the country level, such prioritisation processes can help drive national research funding, prompt updates to AMR national action plans, and support maximal learning from national AMR actions.

#### Systematic reviews

Researchers need to use rigorous systematic reviews to inform research prioritisation and to summarise the effectiveness of policy interventions.

Rigorous systematic reviews and evidence gap maps can support the planning of policy interventions by ensuring that they are adequately supported by evidence. However, to be useful, these reviews must be high quality and regularly updated. Health Systems Evidence has appraised and catalogued more than 50 systematic reviews related to health systems and antimicrobial use dating back to the year 2000, and this database shows that the rigour and quality of these reviews is mixed [39].

Conducting systematic reviews to summarise what we currently know is an essential input for research prioritisation. Reviews can collate empirical evidence to answer a specific research question [40] but they can also map the availability of evidence to identify evidence gaps. The Campbell Collaboration and others have recently developed methodological guidance for creating evidence gap maps [41]. Systematic reviews need to be regularly updated to include new evidence as it arises, to determine whether research gaps have been addressed, and to determine whether the research question has been satisfactorily answered or whether future, more refined research is needed. Given that the current evidence base on AMR policy is weak [6], policy decisions in the near future will be informed by relatively weak signals from the research base, which reinforces the need for further evaluation.

Systematic reviews can also ensure that research efforts are not wasted on questions that have already been definitively answered [23, 42]. Although replication is key to science, there is a point at which additional replication holds little additional value [26]. For example, it has been definitively shown that hospital antimicrobial stewardship interventions are effective at reducing antimicrobial use compared to control and should be a standard part of the AMR response [17]. Future research can, instead, focus on optimising stewardship for different contexts in order to maximise effects. This includes opportunities to embrace a philosophy of radical incrementalism, where a series of small evaluated changes one after the other result in radical cumulative change [43]. Finally, employing advanced analytical techniques, such as network meta-analysis, in systematic reviews can improve research prioritisation by enabling better exploration of heterogeneity in reviews of complex interventions [44].

#### More planned evaluations

Researchers need to work with stakeholders to ensure that rigorous evaluations of all new AMR programmes are the norm.

Researchers can actively advance progress on AMR by ensuring that evaluations of policies become the norm. While rigorous evaluations of all AMR interventions would be ideal, at present, we are very far from this reality [21]. Policy responses to AMR — from legislation and government regulation to public awareness campaigns — have played a major role in responding to AMR, yet these policies are rarely conceptualised as population health interventions and, as a result, are rarely pilot-tested, reviewed or evaluated with sufficient rigour to expand the AMR evidence base.

Without a culture of evaluation, we risk implementing, maintaining and even spreading ineffective or inefficient AMR policies, at great financial and opportunity costs. Additionally, though policy-makers do not always see their added value, there are political advantages in conducting good impact evaluations; evaluation puts policy-makers in the politically attractive position of continuous policy improvement, enables them to ensure that research assessing their initiative is appropriate, and reduces political risk because they can acknowledge that they are operating with imperfect information [45]. Where possible, researchers should advise and partner with policy-makers to raise the rigour of evaluations, simultaneously making progress on scientific questions. In particular, researchers should advocate for the development of protocols and evaluation plans a priori, which will help minimise waste of public resources in ineffective programmes by ensuring that the data collected is appropriate to answer key policy-maker questions, while also supporting implementation and future improvements in practice by ensuring that data is internationally comparable, and can feed into future evidence syntheses of similar policy interventions.

#### Research that is responsive to stakeholder needs

AMR research needs to be planned to address policy-makers’ questions about effectiveness, implementation, costs and equity.

Moving from evidence to policy inevitably involves consideration of pragmatic and ideological factors beyond evidence of effectiveness [46]. Researchers can support evidence-informed policy-making by considering, in advance, the likely information needs of policy-makers. In addition to effectiveness, decisions to pursue or pass over various AMR policy interventions are likely to be informed by their perceived cost-effectiveness, equity and differential impacts based on gender, race and socioeconomic status, implementation challenges, and acceptability to diverse social groups. This is particularly true in the case of LMICs, where resources are scarce and where the level of evidence required before investing in policy action may be substantially higher [45].

One simple and intuitive tool for enabling policy-maker engagement in research planning is to use the APEASE criteria [46] (Box 2). Originally created as a framework for evaluating ideas for interventions, APEASE offers a useful structure for framing research questions and evidence needs in partnership with stakeholders and for communicating policy-relevant research findings. APEASE addresses many common stakeholder concerns, including equality and equity considerations, acceptability across a wide range of groups, and the feasibility and practicality of an intervention in a given context, while recognising important trade-offs. Consider, for example, that both the clinical effectiveness and cost-effectiveness of an intervention is irrelevant if the intervention is unaffordable or infeasible given funding and resource constraints in a specific context. In addition to the feasibility of implementation, it is useful to consider whether the intervention should be, or can be, equally applied across the whole population, and whether it will reach its target population and intended beneficiaries. Again, the inclusion of these additional research questions in the planning phase will particularly benefit policy-makers in lower-resource settings, who must consider whether interventions from high-income settings could translate effectively to their setting, given the staff and resource limitations particular to their context.

### Research conduct

#### Better design of AMR interventions

Researchers need to use theory, frameworks and logic models to design more coherent AMR policy interventions.

Unfortunately, to date, the approach to designing AMR policy interventions has been ad hoc and seems to be guided by the ‘it seemed like a good idea at the time’ principle, rather than by an explicit process that considers the determinants of the problem, relevant theory and available empirical evidence [17, 21]. This strategy has often resulted in poorly considered AMR policies that, although designed to change attitudes, beliefs and practices around antimicrobial use, cannot clearly articulate how their intervention will successfully bring about this change [21]. Poorly designed policies may also fail to recognise and address key AMR determinants, leading to ineffective or sub-optimally effective interventions.

Researchers can improve the design of AMR policy interventions by employing and advocating for the inclusion of theory, frameworks and logic models in the early stages of intervention design to describe how and why an intervention is expected to work. These steps can substantially address the common tendency in AMR to re-invent the ‘square’ wheel rather than build on existing evidence from behavioural and implementation science. When planned without the use of theory, interventions are more likely to be unclear about the behaviours and outcomes targeted, and the means by which the intervention will achieve its intended effect [17]. The process of building such models can encourage researchers to consider all aspects of the intervention and the existing AMR evidence base. The United Kingdom Medical Research Council has published a useful framework for developing and evaluating complex interventions [47, 48]. Other useful frameworks include the Behaviour Change Wheel [46, 49] to guide intervention development, the Theoretical Domains Framework to assess factors that impact behaviour [50], and the Behaviour Change Techniques taxonomy, which considers individual component strategies employed to change behaviour (e.g. feedback on behaviour, goal setting, prompts and cues) under the umbrella of a larger intervention [51]. Finally, the use of theories, frameworks and logic models also facilitate research communication and dissemination, making clear the considerations and circumstances that drove the initial hypothesis [50].

#### Better design of evaluations

Researchers need to ensure that AMR policy interventions are evaluated using the most rigorous designs feasible in the given circumstances.

When promoting a culture of AMR policy evaluation, improving and strengthening the design of evaluations should also be considered. One challenge in AMR research has been a lack of differentiation between programme evaluation (i.e. evaluating whether the local programme achieved its goals) and research evaluation (i.e. addressing generalisable concerns about what, when, how and why an intervention works). While programme evaluation is important, policy-making needs to be guided by robust evidence generated using rigorous study designs. AMR policy intervention research has been plagued by poor quality and inappropriate evaluation methods. In a recent systematic review, 30 of 69 included studies used uncontrolled before–after designs and simple descriptive methods that cannot control for important design concerns, including bias, confounding and secular trends, and are generally uninterpretable [6, 52, 53].

**Fig. 2:**
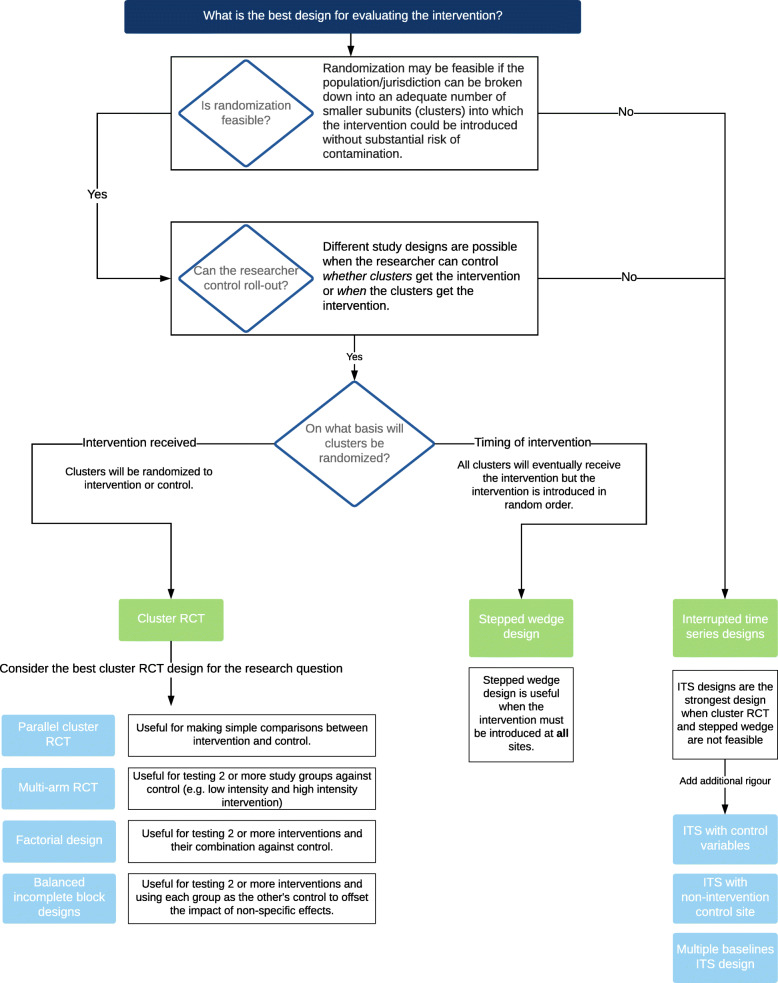
Considerations when choosing a prospective evaluation design for AMR policy interventions

Overall, interventions should be evaluated using the most rigorous study designs feasible in the given circumstances in order to minimise bias and maximise the generalisability of findings. The choice of evaluation design will be guided by numerous factors, such as whether it is feasible to randomise intervention sites, whether it is necessary to introduce the intervention at all sites simultaneously, the acceptability of having a no-intervention control group, and the number of available intervention sites (Fig. 2).

**Fig. 3:**
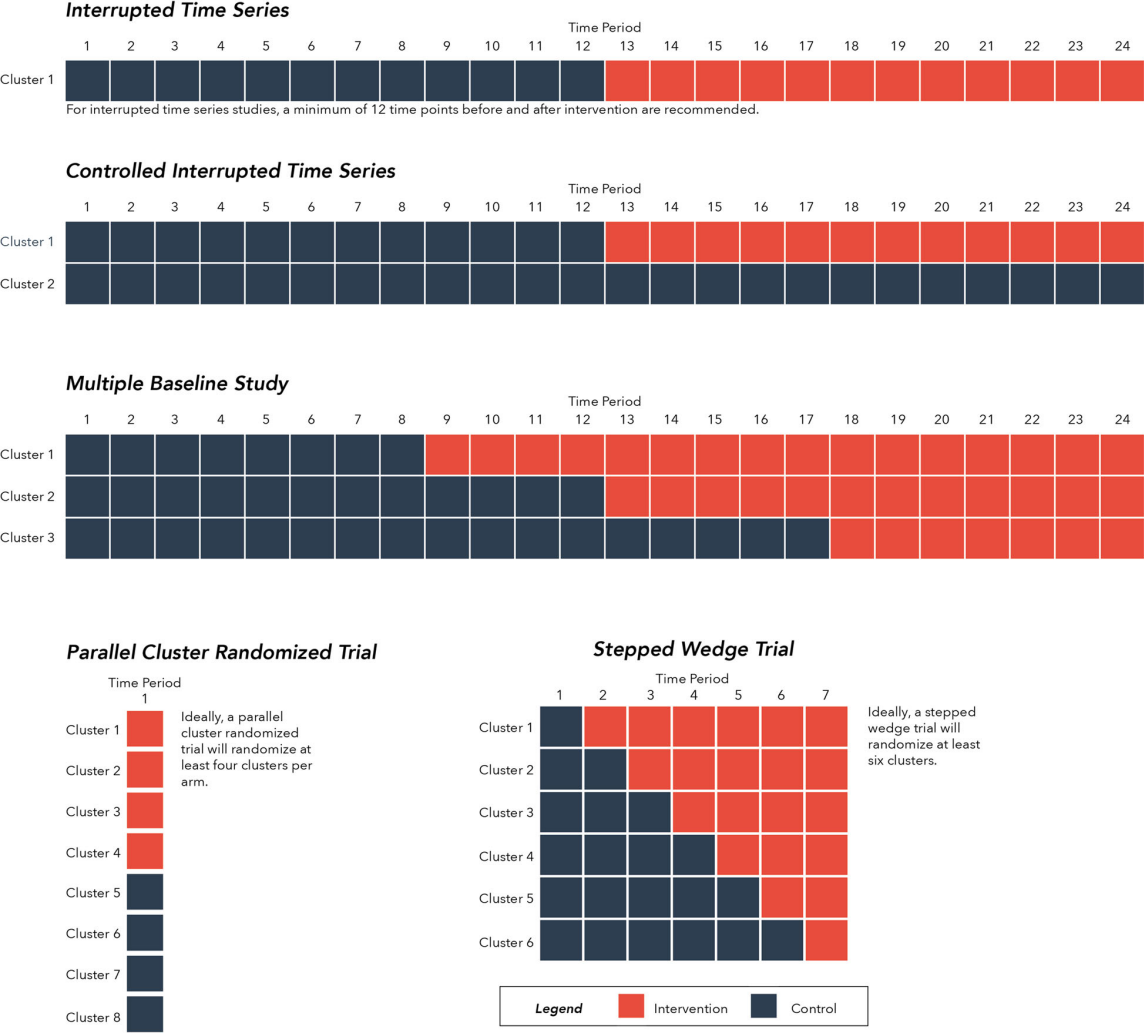
Recommended study designs for evaluating AMR policy interventions

The focus on education, attitudes and behaviour change in AMR research, and the associated high likelihood of contamination, will likely preclude the use of randomised controlled trials (RCTs) with individuals (e.g. citizens, patients) as the units of randomisation in much of AMR policy intervention research. In most cases, a cluster RCT should be considered the gold standard for AMR policy intervention evaluations [54]. Cluster randomisation can be done at numerous levels, although trade-offs exist between the number of available units (e.g. regional level) and the risk of contaminations (e.g. provider level). There are many possible cluster randomised trial designs, including two arm trials, multi-arm trials and factorial designs where two or more interventions can be implemented simultaneously. Another cluster design, the stepped wedge trial — where all sites start in the control arm and end in the intervention arm, crossing over to intervention sequentially and in random order [55] — may have many practical benefits for large policy evaluations where roll-out of an intervention to all sites within a health system or community is a requirement [56]. Figure 3 illustrates these recommended evaluation designs.

Where random allocation is not feasible (either due to an inadequate number of randomisation units being available or because simultaneous implementation across the health system is required) interrupted time series (ITS) methods are the strongest study design for AMR research [57]. However, adding a control group [58] or additional sites with staggered implementation of interventions (multiple baselines ITS design [59]) can strengthen this design. Researchers should pay particular attention to whether an ITS design is appropriate for their study. ITS designs are best used for evaluating AMR interventions that have been implemented at a clearly defined point in time [57]; many AMR studies have inappropriately used this design to measure complex interventions rolled out in stages across several months or years. de Kraker et al. have carefully considered the validity and bias concerns associated with these study designs and have published two excellent guides for evaluating antimicrobial stewardship interventions, which are also highly relevant to other AMR policy interventions [52, 54].

Uncontrolled before–after study designs, which historically have been common in AMR research, should be avoided if at all possible. The apparent effects of an intervention using these designs are completely confounded by secular trends and concurrent events. Even controlled before–after studies, which mitigate this threat of confounding to a limited extent, should be avoided unless there are at least two intervention sites and two control sites; however, given more sites, other more rigorous study designs may be feasible and preferable. Controlled before–after studies and non-randomised trials are an option when randomisation is not possible and there is an insufficient number of time intervals to conduct an ITS; however, these designs should only be considered as hypothesis generating. Figure 2 outlines some of the methodological considerations for choosing a prospective evaluation design.

#### Iterative improvement on existing trials

AMR policy research needs to include head-to-head comparisons of different intervention variations and promote radical incrementalism to enhance the effectiveness of extant policies.

Researchers can advocate for the use of more rigorous study designs and partner with policy-makers to ensure that evaluations are appropriately conducted. To move beyond effectiveness, research evaluations need to incorporate opportunities for addressing the other important AMR policy questions described earlier, namely why interventions work and what elements are necessary for their success. Once the initial effectiveness of an AMR policy intervention has been shown, there is an opportunity to conduct controlled head-to-head comparisons of different intervention iterations in the interest of optimisation, either through sequential trials comparing variations of the intervention, or using factorial designs to evaluate whether the addition of co-interventions meaningfully changes the intervention’s effectiveness. Evaluations can be facilitated by the development of implementation laboratories, which involve close collaboration between research teams and health systems delivering implementation strategies at scale [60]. Trials can also be designed to enhance the generalisability of evaluation by including fidelity sub-studies to determine whether an intervention was delivered and received as designed, mechanistic sub-studies to determine whether the intervention acted through the hypothesised pathways, qualitative process evaluations to investigate experience and acceptability, and economic evaluations [61].

#### A set of standard measures and metrics

Researchers need to define a set of core outcome measures for AMR research that address appropriate antimicrobial prescribing, development of antimicrobial resistance, and cost-effectiveness of policy interventions.

AMR lacks both an agreed-upon metric for evaluating progress and a common system for measuring the scope of the problem. Systematic reviews of interventions to reduce antimicrobial use have shown that researchers use a wide range of prescribing and dispensing-focused metrics [6, 17]. The Tripartite Monitoring and Evaluation Framework [62] recently released by WHO, the Food and Agriculture Organization and the World Organisation for Animal Health, is a useful starting point that suggests many important One Health indicators for AMR; however, this framework lacks detail on operationalising these measures. Researchers can have a significant impact in these ongoing conversations by developing measures that facilitate data sharing; for example, through an agreed minimum dataset for collecting intervention data and a core outcome set of measures to facilitate evaluations. Consideration should also be given as to whether a core outcome set can feed into AMR and antimicrobial use surveillance to facilitate the use of routinely collected data in impact evaluations.

A harmonised set of measures for conducting and evaluating interventions serves three purposes. First, it creates consistency between evaluations that facilitates systematic reviews to inform evidence-informed policy-making. Lack of a shared outcome measure is a common challenge in public health systematic reviews and one which limits the amount of evidence that can be rigorously synthesised [63]. Second, creating a harmonised set of measures offers the opportunity to include, as a requirement, metrics beyond impact that are relevant to stakeholders such as equity considerations and cost-effectiveness. Finally, developing a set of harmonised measures offers an opportunity to consider common barriers to AMR policy implementation and to develop standard measures and indicators that also address these needs.

While there are substantial political and economic barriers to improving data collection and evaluation, from a metrics viewpoint, there are numerous successful initiatives that have adopted research governance principles to improve data collection and comparability of research studies, including the Core Outcome Measures in Effectiveness Trials (COMET) group [64] and ESSENCE for Health Research [65]. Outcome Measures in Rheumatology (OMERACT), for example, uses a data-driven, iterative process to choose shared outcome measures across four domains, and each selected measure must meet three criteria – truth, discrimination and feasibility [66].

### Research dissemination

#### Better reporting of interventions

Researchers need to register research protocols and evaluation trials and use reporting guidelines and checklists to improve reporting quality.

An effective and coherent global response to AMR requires full and transparent reporting of all aspects of AMR intervention studies. Unfortunately, public health research as a whole, and AMR research specifically, faces challenges in this domain. Inaccessible research, partial reporting, and publication bias are all common in AMR research [21, 67] and limit opportunities for researchers and policy-makers to learn from experiences in other contexts.

Researchers should register protocols, primary evaluations of policy interventions (both RCTs and quasi-experiments) and systematic reviews in trial registers. Pre-registration of an evaluation helps address the tendency to avoid publishing the results of research with neutral or negative findings, which is common to much of scientific research, and may be particularly common in policy research where government partners may feel that they lose credibility if a policy intervention is found to be less effective than expected. The fields of clinical trials and systematic reviewing — both of which use protocol registration to reduce publication bias and to enable other researchers to see whether a research study on a particular topic is ongoing — have benefited from the support of research funders, journals and research ethics boards to promote these efforts to improve research reporting [67]. Along with trial registration, researchers, governments and other implementing organisations should commit to full and transparent reporting of study results in open access journals, to making datasets available to other researchers and engaging in knowledge translation.

Researchers should also embrace the use of reporting guidelines and checklists to overcome common reporting challenges such as not describing the intervention specifically enough to allow replication [21, 50], using the same description to represent different types of interventions [68], using different terminology to represent the same content [69, 70], and repetition without improvement [71]. The TIDieR tool [72] for describing and replicating interventions, and its extension TIDieR-PHP [73] for population health and policy studies, are both useful tools for researchers to ensure that their intervention has been thoroughly described. The SQUIRE checklist [74] for quality improvement interventions, the CONSORT statement [75] for RCTs and its many extensions to cluster RCTs [76], stepped wedge trials [56], pilot studies [77], pragmatic trials [78], and the Equator Network [79] are all useful tools to ensure the full reporting of methods and findings within a study report.

#### Shared learning opportunities

Researchers need to embrace open data and open access opportunities to widely disseminate AMR research findings.

The principle of shared learning is familiar to researchers and embedded in much of health research. A key question facing the field is how best to promote this ideal when working with stakeholders to develop effective and efficient AMR policy interventions. However, as many governments embrace nudge units, innovation hubs and radical incrementalism [43], there is an opportunity for researchers to reiterate the substantial benefits of shared learning. Many new methodological tools can support these efforts – data sharing platforms (e.g. the World Wide Antimalarial Resistance Network, WWARN [80]) and open-access information repositories will both go a long way to ensuring the evidence generated from policy experiments and intervention evaluations can support shared learning. Likewise, living systematic reviews, which are continually updated as new evidence is generated [81], will also help ensure that new policies are planned based on current evidence.

**Table:** Box 1

Current state of the evidence base on AMR policy interventions	
Around the world, 129 governments are currently in the process of developing or implementing a National Action Plan to address antimicrobial resistance [19] Global capacity for AMR surveillance is lacking; discrepancies between methods and monitoring systems, data quality concerns and lack of representativeness make it challenging to compare AMR data between countries [20] Many evaluations of AMR policy interventions are conducted retrospectively by academics who were not involved in the design or implementation of the intervention [6, 21] A systematic review of experimental and quasi-experimental studies evaluating government policy interventions to reduce the use of antimicrobials [6, 22] found that 30 of the 69 studies used low-quality study designs, such as uncontrolled before–after designs, which severely limits the validity of their findings. Among these 69 studies, only 4 used a randomised controlled design which is considered the gold standard for evaluating interventions. Another systematic review of 221 interventions for improving antibiotic prescribing among hospital inpatients found the quality of the reporting for the 163 non-randomised studies was so poor that it was difficult for professionals to use the research findings or to implement interventions that were shown to be useful; further, this systematic review found that no useful evidence could be gleaned from studies using controlled before–after and non-randomised trial designs [17] Reporting of AMR policy intervention studies is weak; studies often fail to describe the intervention in sufficient detail for replication and many do not report the reason the intervention is expected to work [21] In the broader field of public health, researchers have estimated that at least 50% of published research is not sufficiently clear, complete or accurate for others to interpret or use [23, 24] There are no standardised measures and metrics for AMR research; many AMR intervention studies report antimicrobial use in defined daily dose per 1000 population or a simple prescribing rate [21]

**Table:** Box 2

The APEASE Criteria [42, 46]	
**A**ffordability **P**racticability **E**ffectiveness/cost-effectiveness **A**cceptability – public, professional, political **S**ide-effects/safety **E**quality	

## Conclusions

Although the threat posed by AMR has been well-known for many decades, recent escalation in multidrug-resistant and extensively drug-resistant bacterial infections has elevated AMR to a more prominent position on the international political agenda. Substantial work is ongoing at the national level to address domestic AMR concerns [19], and there are robust conversations at the international level about pursuing more large-scale, coordinated efforts to mitigate the AMR threat such as an international legal treaty on AMR [8, 11, 82–84]. Innovative approaches have been taken to research funding (e.g. JPIAMR [85]), health systems strengthening (e.g. Accreditation Canada [86]), and global policy monitoring (e.g. WHO [19]).

These national and international efforts require substantially more evidence for the effectiveness and feasibility of AMR policy interventions than is currently available. However, this gap provides an opportunity for researchers to engage in a meaningful conversation about the importance of evidence-informed policy-making for AMR. Mitigating the threat posed by AMR will also require substantial collaboration among researchers and policy-makers. Table 1 describes many of the ways in which research funders, publishers and policy-makers can jointly support researchers and facilitate action across the priorities identified in this framework. With their ability to strategically fund innovative research, encourage researchers to use rigorous study designs and reporting checklists, and facilitate shared learning, these partners have an opportunity to amplify researchers’ calls for better AMR practice.

**Table 1:** Framework and recommendations for planning, conducting and disseminating evaluations of AMR policy interventions

	**Researchers**	**Policy-makers**	**Research funders**	**Publishers**
**Mechanisms for prioritising research**	Prioritise research to study what works, when it works, why it works, and what elements are necessary to its success	Engage with the research community and make your evidence needs clear	Funding support for formal prioritisation processes Targeted grant competitions to drive research in priority areas	Require that research reports summarise the evidence that was already known on a topic and show systematic review evidence that supports the conduct of an intervention
**Systematic reviews**	Inform interventions using rigorous systematic reviews	Partner with researchers to do evidence syntheses to inform policy-making	Strategic funding support for systematic reviews, evidence syntheses, living systematic reviews Require evaluations to be justified by systematic reviews	Support the publication of systematic review protocols, systematic reviews, evidence syntheses and living systematic reviews
**More planned evaluations**	Work with other stakeholders to make rigorous evaluations of all AMR programmes the norm	Working with researchers, plan the evaluation strategy for a programme or policy before launching the programme or policy	Strategic funding support for evaluations of policy interventions and for policy-research partnerships to facilitate this research	Require authors to register study protocols
**Research that is more responsive to stakeholder needs**	Ensure that research addresses all informational needs of policy-makers (e.g. including equity and cost-effectiveness)	Decide in advance what information and evidence you need to inform policy-making	Encourage integrated knowledge translation and collaboration with stakeholders when awarding grants to support AMR policy research	Ensure timely peer review and publication of research to ensure that evidence is available to support stakeholders
**Better design of AMR Interventions**	Use theory, frameworks and logic models in the intervention design phase to frame how and why an intervention is expected to work	Use theory, frameworks and logic models when planning policy interventions to clarify how and why an intervention is expected to work	Do not fund interventions that do not employ theory, frameworks or logic models to describe how and why the intervention is expected to work	Require authors to report on their use of theory, frameworks, and logic in the design and conduct of AMR interventions
**Better design of evaluations**	Use the most rigorous possible evaluation designs to minimise bias and maximise generalisability	Embrace research evaluation to understand what, when, why and how and intervention works	Studies using weak study designs (e.g. uncontrolled before and after designs) should not be funded	Refrain from publishing studies that use poor quality methods such as uncontrolled before and after studies for evaluation of AMR interventions
**Iterative improvement on existing trials**	Conduct head-to-head comparisons of intervention variations	Promote radical incrementalism (based on rigorous evaluation) to enhance the effectiveness of extant policies	Provide funding support for head-to-head trials	Publish research with neutral and negative results
**A set of standard measures and metrics**	Develop a set of core outcome measures for AMR research	Partner with researchers to ensure that core outcome measures address your key evidence needs	Funding support for the development of an AMR core outcome set Require use of core outcome measures in funded applications	Require researchers to use the core outcome measures in published evaluations
**Better reporting of interventions**	Commit to full and transparent reporting of studies Use reporting guidelines and checklists to fully report a study Register intervention protocols to reduce the risk of publication bias Avoid ‘spin’ especially with weak evaluative designs	Publish or make available reports on the effectiveness of policy interventions and efforts to improve them	Make public the details of funded interventions Require full and transparent reporting of studies Require researchers to register the protocols of their interventions	Require authors to use the relevant research reporting guidelines and checklists Publish research with neutral and negative results
**More opportunities for shared learning**	Disseminate research widely and embrace open data and open access opportunities Make datasets available to other researchers through data repositories Develop cross programme collaborations to encourage learning and efficient knowledge generation	Take advantage of opportunities to borrow and adapt policy interventions from other contexts Make available data on policy interventions in your setting to promote uptake in other contexts and ensure that ineffective policy is not duplicated in other settings	Provide funding for open access publishing, open data-sharing platforms, cross programme collaborations and living systematic reviews	Increased commitment and support for open access publication

*AMR* antimicrobial resistance

As we have highlighted, it is increasingly important to strengthen the scientific evidence base on AMR policy interventions, to learn from existing policies and programmes, and integrate scientific evidence into the global AMR response [6]. This framework offers a path forward, increasing local and global cooperation, and overcoming common limitations in existing research on AMR policy interventions.

## Data Availability

Not applicable.

## Abbreviations

AMR: antimicrobial resistance
ITS: interrupted time series
JPIAMR: Joint Programming Initiative on AMR
LMICs: low- and middle-income countries
RCT: randomised controlled trial
